# Benefits of Zebrafish Xenograft Models in Cancer Research

**DOI:** 10.3389/fcell.2021.616551

**Published:** 2021-02-11

**Authors:** Xingyu Chen, Yongyun Li, Tengteng Yao, Renbing Jia

**Affiliations:** ^1^Department of Ophthalmology, Ninth People's Hospital, Shanghai Jiao Tong University School of Medicine, Shanghai, China; ^2^Shanghai Key Laboratory of Orbital Diseases and Ocular Oncology, Shanghai, China

**Keywords:** zebrafish model, tumor visualization, pharmacokinetics, tumor microenvironment, tumor model

## Abstract

As a promising *in vivo* tool for cancer research, zebrafish have been widely applied in various tumor studies. The zebrafish xenograft model is a low-cost, high-throughput tool for cancer research that can be established quickly and requires only a small sample size, which makes it favorite among researchers. Zebrafish patient-derived xenograft (zPDX) models provide promising evidence for short-term clinical treatment. In this review, we discuss the characteristics and advantages of zebrafish, such as their transparent and translucent features, the use of vascular fluorescence imaging, the establishment of metastatic and intracranial orthotopic models, individual pharmacokinetics measurements, and tumor microenvironment. Furthermore, we introduce how these characteristics and advantages are applied other in tumor studies. Finally, we discuss the future direction of the use of zebrafish in tumor studies and provide new ideas for the application of it.

## Introduction

The implantation of tumors into zebrafish has become an emerging model platform in cancer studies. The implanted cancer cells include patient-derived primary cells and laboratory cell lines. Traditional patient-derived xenografts (PDXs) are established from tumor cells or masses isolated from patients during biopsy or excision and are implanted into a model animal, such as immunodeficient mice. To some extent, PDXs retain the heterogeneity of the patient's primary tumor and possess a stable biological profile as it relates to gene expression and mutation (Hidalgo et al., 2014). Mounting evidence has indicated that PDX models maintain the biology of the tumor and can predict the efficacy of drugs based on direct comparisons of drug efficacies in patients and in mice inoculated with corresponding xenografts (Tentler et al., 2012; Siolas and Hannon, 2013; Hidalgo et al., 2014; Rosfjord et al., 2014). Over the past 40 years, mouse patient-derived xenografts (mPDXs) have played an unparalleled role in preclinical trials and personalized medicine. However, the primary issue for their widespread clinical application is the large number of tumor samples required and the time needed for the xenograft to grow. Currently, zebrafish patient-derived xenograft (zPDX) models have elicited increasing attention from researchers in cancer fields because these models can also provide valuable information on the molecular biology of tumors and personalized therapeutic choices for patients with diverse cancer types (Bentley et al., 2015; Lin et al., 2016; Fior et al., 2017; Gaudenzi et al., 2017).

Cancer cells implanted into zebrafish are derived from various types of malignancies, such as germ cell tumors, leukemia, lymphoma, melanoma, sarcoma, neuroblastoma, and various epithelial cancers originating from the neuroendocrine system (i.e., liver, gastrointestinal tract, colon, pancreas, prostate, lung, ovary, and breast) (Haldi et al., 2006; Marques et al., 2009; Weiss et al., 2009; Zhao et al., 2009; Corkery et al., 2011; Latifi et al., 2011; Ghotra et al., 2012; He et al., 2012; Smith et al., 2013; Chapman et al., 2014; Vaughan et al., 2015; Gaudenzi et al., 2017; Lin et al., 2019; Wong et al., 2019).

Regarding its application, the zebrafish model is a high-throughput and low-cost animal model for predicting the efficacy of personalized cancer therapy and exploring mechanisms that drive tumor growth, metastasis, and responses to therapy (Keller and Murtha, 2004; Patton et al., 2005; Zon and Peterson, 2005; Barros et al., 2008; Park et al., 2008; Marques et al., 2009; Smith et al., 2010; Eguiara et al., 2011; Nguyen et al., 2012; Chen et al., 2014; Tang et al., 2014; Mandelbaum et al., 2018; Wong et al., 2019). Zebrafish are tiny and can hatch 150–200 eggs every week, which meets the high-throughput and low-cost requirements for a viable animal model. The zebrafish model can not only be applied to study tumor angiogenesis, cell invasiveness and drug responses in a time- and cost-effective manner but also serve as a real-time *in vivo* platform for personalized cancer treatment (Wu et al., 2017; Rebelo de Almeida et al., 2020). In addition, the zebrafish genome project revealed sequence conservation of multiple genes and identified zebrafish orthologs for 82% of genes related to human disease, suggesting a high interspecies conservation of molecular pathways between zebrafish and humans (Chen and Fishman, 1996; Granato and Nusslein-Volhard, 1996; Howe et al., 2013; Tulotta et al., 2016).

However, there are also limitations and challenges of using zebrafish as a model in cancer research, including inconsistencies with generating models, high mortality after injection, disparity in protocols among laboratories, different body temperatures between fish and humans, and poor reproducibility. Despite these shortcomings, zebrafish are still being applied as a promising model in tumor research.

**Figure 1:**
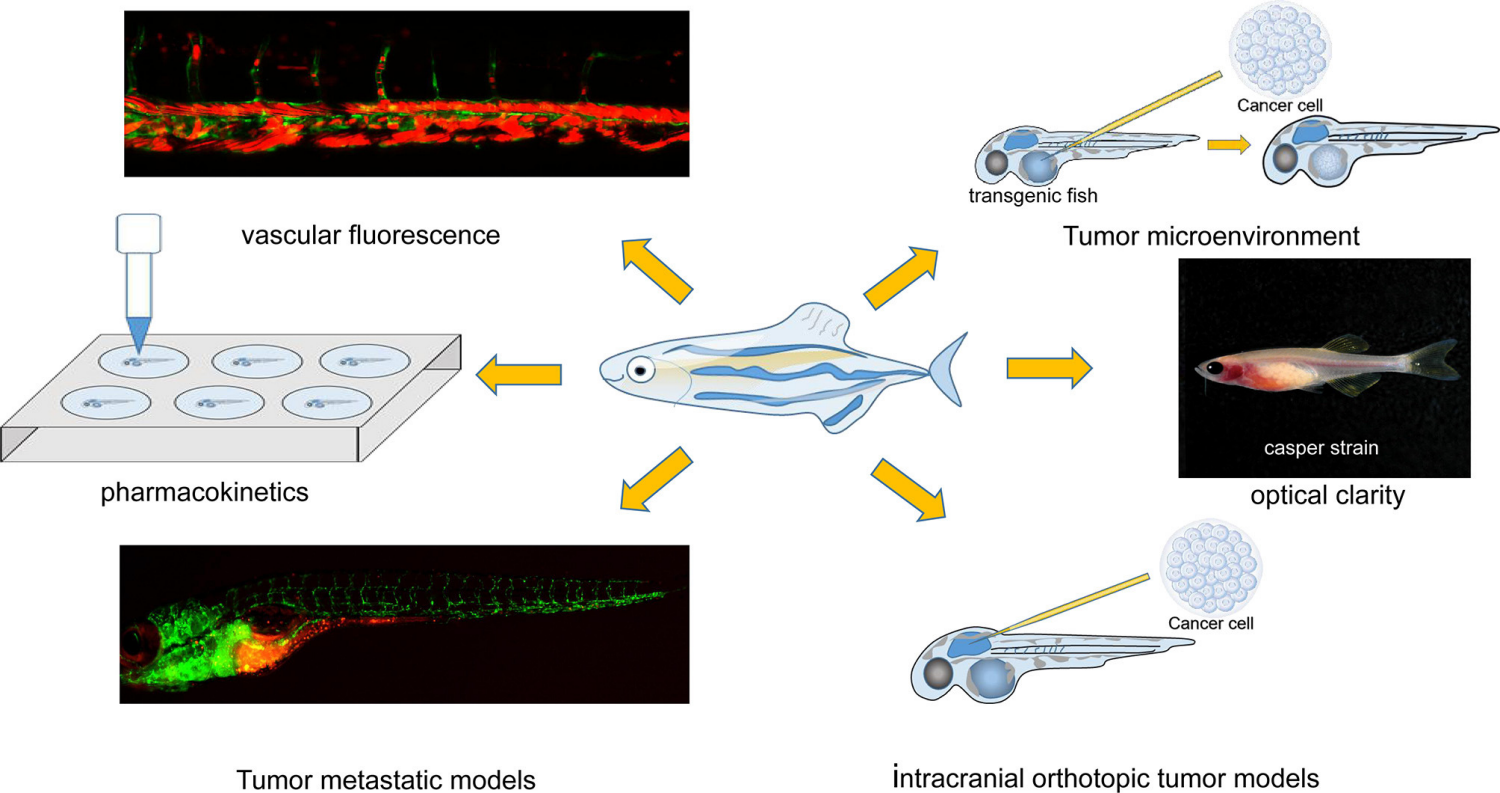
Characteristics of the zebrafish xenograft model. The image of the “Casper strain” was cited from website https://www.carolina.com/.

In this review, we focus on applications of the zebrafish model of implanted tumors to facilitate cancer studies and predict the efficacy of personalized cancer treatment. Characteristics of the zebrafish model, such as the establishment of various tumor models, antitumor drug pharmacokinetics, and the tumor microenvironment (TME) (all of which are of special interest in cancer research), are discussed in detail (Figure 1).

## Classic Characteristics of the Zebrafish Xenograft Model

A tumor model can be created in both embryonic and adult zebrafish. There is currently an immune-deficient adult zebrafish strain that lacks T, B, and natural killer (NK) cells and can be used to establish xenograft models for studying new therapies (Traver et al., 2003; Langenau et al., 2004; Patton et al., 2005; Stoletov et al., 2007; Blackburn et al., 2012; Tchoghandjian et al., 2012; Tang et al., 2014; Moore et al., 2016; Drabsch et al., 2017; Yan et al., 2019). For instance, optically clear *prkdc*^−^/^−^, *il2rga*^−^/^−^ transgenic zebrafish, which lack an adaptive immune system and NK cells, can be engrafted with human cancer cells at 37°C (Yan et al., 2019; Costa et al., 2020a). If the xenograft is transplanted at early embryonic stages [blastula stage to 48 h postfertilization (hpf)], at which point the adaptive immune system has not yet matured, the procedure does not require immune suppression (Lam et al., 2004; Haldi et al., 2006; Nicoli et al., 2007; Konantz et al., 2012; White et al., 2013). It has been reported that cancer cells labeled with a fluorescent marker (such as CM-DiI) and injected into 48-hpf embryos can be tracked for more than 7 days post fertilization (7 dpf) (White et al., 2013; Wu et al., 2017). During this period, the size and shape of the implanted tumor can be evaluated by fluorescence microscopy, confocal microscopy, pathological biopsy, and other techniques (Pontes et al., 2017).

Other transgenic zebrafish strains, such as the “*Casper*” line (*roy*^−/−^;*nacre*^−/−^) and vascular-specific green fluorescent protein (GFP) strains, might be considered useful tools for other cancer-related studies (Zon and Peterson, 2005; Haldi et al., 2006; Mizgireuv and Revskoy, 2006; Marques et al., 2009; Stoletov et al., 2010; Eguiara et al., 2011; Zhao et al., 2011a,b; D'Agati et al., 2017; Pontes et al., 2017; Lin et al., 2019). The pigment-deficient “*Casper*” strain provides optically clear visualization to observe xenograft cancer cells (White et al., 2008; Cho et al., 2015; Heilmann et al., 2015; Li et al., 2015; Tang et al., 2016; D'Agati et al., 2017). The transparency of the zebrafish body makes it possible to track two or more types of cells labeled with different fluorescent colors and explore their interaction (Corkery et al., 2011; Liu and Leach, 2011; Wang et al., 2015; Vazquez Rodriguez et al., 2017; Sun et al., 2019). The “*Casper*” strain is also useful for tracking extracellular vesicles (EVs) released from tumors and dissecting their role in forming the metastatic niche *in vivo* (Hyenne et al., 2019).

Tumor neovascularization is essential to the initiation, progression, and invasion of cancers (Folkman, 1971, 1989; Chung et al., 2010; Jászai and Schmidt, 2019). Exploration of the mechanism of neovascularization in tumors and the development of antiangiogenic agents to inhibit tumor neovascularization are two focuses of tumor therapy studies (Folkman, 1989; Bergers et al., 1999). The Tg(*fli*:eGFP) zebrafish line with GFP-labeled vasculature contributes largely to studies on the mechanism of tumor neovascularization and evaluations of the efficacy of antiangiogenic agents (Tran et al., 2007; Fior et al., 2017; Gaudenzi et al., 2017; Britto et al., 2018, 2019; Cirello et al., 2018; Garcia-Caballero et al., 2018). For instance, researchers directly observed and compared the *in vivo* conditions needed to create GFP-positive vessels in the zebrafish strain Tg(*kdrl*:eGFP)s843, a transgenic zebrafish line expressing eGFP under the control of the endothelial *kdrl* promoter. These models are grafted with normal DU-145 prostate cancer cells and transfected with four microRNAs (miR-125a, miR-320, miR-487b, and miR-492) (Chiavacci et al., 2015). Studies have concluded that angiogenic growth factors are locally released by angiogenic tumor cells, and the microRNAs applied can inhibit this process (Chiavacci et al., 2015). The Tg(*fli*:eGFP) zebrafish line is also used to test the effect of drugs on blocking or preventing tumor neovascularization (Franich et al., 2020).

Although zebrafish are not mammals, they have several tissues, organs and glands whose functions are similar to those of mammals; these include the musculoskeletal and cardiovascular systems, eyes, brain, liver, heart, gastrointestinal tract, and pancreas (Langenau et al., 2003, 2007; Ober et al., 2003; Patton et al., 2005; Sabaawy et al., 2006; Park et al., 2008; Lee et al., 2009; Gutierrez et al., 2011; Jung et al., 2011; Neumann et al., 2011; Rudner et al., 2011; Li et al., 2012; Nguyen et al., 2012). The orthotopic zebrafish tumor model has emerged in studies of multiple types of cancers, such as gastrointestinal tumors, glioblastoma, pancreatic cancer, retinoblastoma, and uveal melanoma (Marques et al., 2009; Lal et al., 2012; Jo et al., 2013; Rampazzo et al., 2013; van der Ent et al., 2014a, Chen et al., 2015; Eden et al., 2015; Hamilton et al., 2016; Jung et al., 2016; Vittori et al., 2016; Welker et al., 2016). Researchers have utilized zebrafish for the orthotopic transplantation of rhabdomyosarcoma to open new avenues for personalized therapy (Yan et al., 2019). The orthotopic tumor model can simulate real tumor conditions in humans; particularly, orthotopic models of brain tumors in zebrafish have been reported and will be further described. The applications of the zebrafish xenograft model are summarized in Table 1.

**Table 1:** Application of the zebrafish xenograft model.

**The tumor source**	**Label**	**Injected object**	**Location of injection**	**Detecting instrumentation**	**Observed indicator**
Patient-derived primary cells Bentley et al. (2015), Lin et al. (2016), Gaudenzi et al. (2017) Fior et al. (2017)	Fluorescent dye (such as CM-Dil) Ghotra et al. (2012), Wertman et al. (2016)	Adult zebrafish Tang et al. (2014), Zhang et al. (2018)	Subcutaneous injection Zhang et al. (2018)	Confocal microscopy/microscopy (Pontes et al., 2017)	Tumor proliferation (Fior et al., 2017; Gamble et al., 2018)
Laboratory human cell lines Lin et al. (2019), Wong et al. (2019), Smith et al. (2013), Chapman et al. (2014), Haldi et al. (2006), He et al. (2012), Corkery et al. (2011), Marques et al. (2009), Weiss et al. (2009), Latifi et al. (2011), Zhao et al. (2009), Ghotra et al. (2012), Vaughan et al. (2015)	Fluorescent protein (such as GFP) Yan et al. (2019)	Embryonic zebrafish (such as 48-hpf embryos) Ghotra et al. (2012), Pruvot et al. (2011)	Orthotopic injection (such as brain) Vittori et al. (2016), Lal et al. (2012), Eden et al. (2015), Wenger et al. (2017), Banasavadi-Siddegowda et al. (2018), Gamble et al. (2018), Casey et al. (2017)	Fluorescence microscopy (Pruvot et al., 2011; Ghotra et al., 2012)	Cell differentiation (Rampazzo et al., 2013)
Laboratory zebrafish cell line Zhang et al. (2018)			Yolk sac Ghotra et al., (2012), Letrado et al. (2018), Wertman et al. (2016)	Histology (Welker et al., 2016)	Cell interaction (Wang et al., 2015; Vazquez Rodriguez et al., 2017; Fornabaio et al., 2018; Labernadie and Trepat, 2018)
			Perivitelline space Nicoli et al. (2007), van der Ent et al. (2014a)	BLI (bioluminescence imaging) (Zhao et al., 2009)	Angiogenesis (Fior et al., 2017; Gamble et al., 2018)
				MRI (magnetic resonance imaging) (White et al., 2008)	Invasion/metastasis/ adhesion (Ghotra et al., 2012; Konantz et al., 2012; Follain et al., 2018; Gamble et al., 2018; Fenizia et al., 2019)
				PET imaging (positron emission tomography) (White et al., 2008)	Cell status (Yan et al., 2019)
				Ultrasound microscopy (Goessling et al., 2007)	Fluorescently labeled nanoparticles/ biodistribution (Sieber et al., 2017; Chen et al., 2019)
				Epifluorescence imaging (Dumartin et al., 2011)	Hemodynamic status (Follain et al., 2018)

## The Zebrafish Model as a Metastatic and Intracranial Orthotopic Tool in Cancer Research

Tumor metastasis models and intracranial orthotopic tumor models are essential in cancer research. In this regard, the zebrafish tumor model can also mimic tumor development in the human body.

Cancer metastasis and recurrence remain the most significant threats to patients (Yan et al., 2019). For some particular cancer types, including uveal melanoma, Ewing's sarcoma and triple-negative breast cancer, the use of metastatic models is preferred over orthotopic models (Paulussen et al., 1998; Tomasin et al., 2019; Johansson et al., 2020). Once the clinical symptoms of these types of tumors, such as a palpable mass, arouse the patient's attention, distant metastases have likely already occurred (Paulussen et al., 1998; Tomasin et al., 2019; Johansson et al., 2020). Therefore, metastatic animal models need to be established to evaluate the ability of cancer cell migration and the efficacy of antitumor drugs.

### Tracking Cancer Cell Intravasation

Currently, metastasis of human cancers is largely studied via transgenic and immunocompromised mPDX models, which are useful for measuring the end-point tumor volume but are unable to quantify or recapitulate *in vivo* invasion, intravasation, extravasation, or the secondary tumor growth of patient-derived cancer cells in real time (Morton and Houghton, 2007; Konantz et al., 2012; van der Ent et al., 2014b; Barriuso et al., 2015; Hill et al., 2018; Yan et al., 2019). The methods used to establish an mPDX metastasis model include cell injection into the tail vein, spleen, or left ventricle, but the key step of metastasis, cancer cell intravasation, cannot be simulated (Goddard et al., 2016; Lizardo and Sorensen, 2018). Zebrafish, however, can offer many advantages as a model system for studying the complex, multistep processes involved during spontaneous cancer metastasis (Fior et al., 2017; Follain et al., 2018; Hill et al., 2018; Wong et al., 2019). Implantation into the yolk sac, perivitelline space (PVS) and subcutaneous tissues allow the direct injection of cancer cells into the zebrafish's body (Abercrombie and Ambrose, 1958; Pruvot et al., 2011; Canella et al., 2017; Letrado et al., 2018). The yolk sac implantation method is more often used by researchers because it is a practical method that accommodates more cells (Wertman et al., 2016; Letrado et al., 2018). For example, by employing *in vivo* imaging coupled with 3D reconstruction and time-lapse microscopy, researchers can monitor interactions between cancer cells and the external surface of zebrafish vessels and record the process of tumor cells migrating along the vascular tubules in addition to morphological changes during the process (Fornabaio et al., 2018). Similarly, the zebrafish model can be used to research the morphological characteristics of tumor cells and the interactions between tumor cells and other cells (Vazquez Rodriguez et al., 2017; Labernadie and Trepat, 2018). However, the primary limitation of this method is that the yolk sac is composed of bulk proteins or lipids that differ from those of the patient's tumor microenvironment and that affect the phenotype and survival of tumor cells (Canella et al., 2017; Sant and Timme-Laragy, 2018). Comparatively, subcutaneous injection in the PVS provides a more beneficial tumor microenvironment for tumor implantation, cell survival, angiogenesis and metastasis (Zhao et al., 2011a; Fior et al., 2017; Costa et al., 2020b).

### Relationship Between Hemodynamics and Metastasis

By using an easy-to-use angiographic zebrafish model, researchers can illuminate the relationship between hemodynamics and tumor formation via circulating tumor cells (CTCs). In the zebrafish model, researchers have found that blood flow is essential for endothelial remodeling and subsequent prometastatic extravasation, even though slow blood flow allows the arrest and adhesion of CTCs. Furthermore, researchers have observed that laminar flow is conducive to the arrest of CTCs and stimulates endothelial remodeling. The same report also suggested that the key to successfully treating metastatic tumors is interfering with endothelial remodeling (Follain et al., 2018). It is worth noting that individual cancer cells can be dynamically visualized in the zPDX model, especially during metastasis (Hill et al., 2018; Astell and Sieger, 2019; Yan et al., 2019).

### Monitoring the Behavior of Single Cancer Cells in Metastasis

Patient-derived tumors have higher heterogeneity than do laboratory-stable tumor cell lines (Fior et al., 2017). Furthermore, patient-derived cancer cells possess certain properties of stem cells that laboratory tumor cell lines do not always offer, including those derived from Wilms' tumor, rhabdomyosarcoma and osteosarcoma (Hirschmann-Jax et al., 2004). It is necessary to study the behavior of a single cancer cell; however, to date, the visualization of a single cancer cell in the mPDX model remains highly challenging and requires a complex, accurate, multistep operation process to successfully implement (Ellenbroek and van Rheenen, 2014). Using confocal microscopy, studies can dynamically observe and record the single-cell behavior of cancer cells either expressing GFP or labeled with a fluorochrome (Follain et al., 2018; Hill et al., 2018; Shi et al., 2019; Yan et al., 2019). For instance, researchers analyzed the behavior of a single cancer cell *in vivo* in a zPDX model and concluded that rhabdomyosarcoma (RMS) cells are composed of three cancer cell types: (1) highly migratory cells, (2) actively proliferating cells, and (3) bystander cells (Ignatius et al., 2012, 2017; Yan et al., 2019). Confocal microscopy can also be used to observe self-renewal, cell-state transitions, regeneration and the other hallmarks of cancer at the single-cell level in the xenograft setting (Moore et al., 2016). Furthermore, the Tg(*fli*:eGFP) zebrafish line with GFP-labeled vasculature allows the direct study of the relationship of vascular endothelial cells with cancer cell intravasation and extravasation and accurately identify the position of the cancer cells (Weiss et al., 2009; Gill et al., 2010).

### Evaluation of The Metastatic Ability of a Cell Line *in vivo*

To test whether the expression of different genes in tumor cells affects metastasis *in vivo*, we need an animal model in which tumors easily metastasize. For example, using an embryonic zebrafish model, researchers have verified that upregulated CD133 expression causes BAK-R cells to be more invasive and metastatic than BAK-P cells (Simbulan-Rosenthal et al., 2019).

The advantages of the zebrafish model are shown not only when studying tumor metastasis but also when establishing orthotopic intracranial tumors. From an structural point of view, the brain morphology in zebrafish is similar to that in humans. Zebrafish also have brain structures that are homologous to those of humans, such as the optic tectum, thalamus and cerebellum (Guo, 2009). However, in contrast to humans, zebrafish manifest equivalent parts of the brain stem in the most dorsal positions of the telencephalon (Wullimann and Mueller, 2004; Leung et al., 2013; Smith et al., 2013). Hence, orthotopically transplanted tumors at this position can be easily established with less damage to cerebral tissue in the zebrafish model (Lal et al., 2012; Eden et al., 2015; Vittori et al., 2016; Casey et al., 2017; Wenger et al., 2017; Banasavadi-Siddegowda et al., 2018; Gamble et al., 2018). Additionally, the blood–brain barrier (BBB) is a specialized tissue originating from brain capillaries that restricts the flow of various molecules, drugs and cells into and out of the brain (Levin et al., 1984, 2015; Neuwelt, 2004; Banks, 2009; Abbott et al., 2010; Krueger and Bechmann, 2010; Ago and Kitazono, 2014; Attwell et al., 2016; Moore et al., 2016). It has been reported that the BBB of zebrafish and mammals are highly similar with regard to formation and function (Jeong et al., 2008; Kulkarni et al., 2014; Moore et al., 2016); therefore, researchers can simulate and observe the process of cancer cell metastasis across the BBB in the zPDX model and explore the mechanism(s) involved (Tanner, 2018).

## The Zebrafish Model in Anticancer Therapy Studies

The zebrafish model has been widely adopted as an emerging anticancer screening platform. Its distinguishing features include rapid turnaround, small space requirements, small sample size requirements, low doses of screened drug, and high-throughput capability (Veinotte et al., 2014; Hamilton et al., 2018; Cornet et al., 2019; Cully, 2019; Hason and Bartuněk, 2019; Yang et al., 2019). Although many zebrafish can be produced after a single spawning event, they can be numbered and tracked individually, which makes the experimental results more accurate. Additionally, since the zebrafish model is prone to metastasis, we can also observe the inhibitory effect of therapy on tumor metastasis during drug screening.

Despite the widespread use of mouse models, researchers have found a minor shortcoming of mouse models of implanted tumors: pharmacokinetics. Rapid liver clearance in mice fails to maintain a stable blood drug concentration and prevents us from adequately assessing drug efficacy (Frapolli et al., 2006; Stuurman et al., 2013; Herbrink et al., 2015). However, the zebrafish model compensates for this limitation. For example, a previous study revealed that adipocytes increase the proliferation and invasion of adjacent melanoma cells. Upon directly injecting lipofermata, which abrogates lipid transport into melanoma cells, into tumor tissue in a zebrafish model, researchers observed a significant decrease in melanoma growth (Zhang et al., 2018). This process was accompanied by an obvious decrease in the lipid content of medically treated tumors, which confirms that lipofermata is effective without undergoing rapid liver clearance (Zhang et al., 2018).

The reason why zebrafish can serve as an ideal model for pharmacokinetic studies is their habitat. Zebrafish live in water and can rapidly absorb chemicals from the aqueous environment across the gills and buccal cavity. Drug screening is easy because researchers can directly add the drug to the water (Lieschke and Currie, 2007; Wang et al., 2010; Wu et al., 2017; Garcia-Caballero et al., 2018). This method has the following pharmacokinetic advantages: (1). the drug concentration *in vivo* in zebrafish can be continuously replenished in the aqueous environment; and (2). drugs in water can penetrate into subcutaneously implanted tumors (Zhang et al., 2015; Garcia-Caballero et al., 2018).

Currently, nanocarriers containing antitumor drugs have become a research focus because prolonging blood drug concentrations increases drug efficacy and decreases adverse drug effects via enhanced permeability and retention effects (EPRs) in solid tumors. Hence, the optimization of pharmacokinetics and biodistribution plays an essential role in the efficacy of nanomedicines. Zebrafish, as a nanomaterial drug delivery system screening tool, presents unique advantages such as a high level of genetic homology to humans, availability of transgenic lines and, most importantly, optical transparency (Lieschke and Currie, 2007; Delvecchio et al., 2011; Sieber et al., 2017). For example, transgenic zebrafish lines that selectively express fluorescent proteins in specific tissues, such as the vasculature, liver and spleen, allow the observation of the biodistribution of fluorescently labeled nanoparticles and the exploration of the circulatory behavior of nanoparticulate drug delivery systems (Gong et al., 2013; Evensen et al., 2016). These zebrafish strains can also allow researchers to directly observe the tumor-targeting effect of the drug. For example, zebrafish xenografts have been chosen as a rapid screening platform for testing the efficacy of monoclonal antibodies in cancer therapy, such as cetuximab and bevacizumab. The result indicates a similar behavioral response to therapies in patients as in zPDXs. It has been suggested that zebrafish xenografts are a promising *in vivo* screening platform for precision medicine (Fior et al., 2017; Rebelo de Almeida et al., 2020). As mentioned above, zebrafish provide a useful model to study the restrictive ability and permeability of the BBB (O'Brown et al., 2019). In the same manner, researchers can observe and explore the encephalic distribution and concentration of anticancer drugs to provide a basis for the treatment of intracranial tumors (Zeng et al., 2017). Researchers can also directly quantitatively evaluate the toxic effects of antitumor drugs in the zebrafish model (Raeber et al., 2005; Fisher, 2011; Junttila and de Sauvage, 2013; Damhofer et al., 2015; Cavalloni et al., 2016; Qin et al., 2016). For example, embryonic death and abnormal phenotypes such as pericardial edema, retarded swim bladder inflation, spinal curvature, short body length, and other adverse effects of drugs can be directly observed only via optical microscopy (Gutiérrez-Lovera et al., 2019; Lin et al., 2019; Vicente et al., 2020). Small-molecule inhibitors can block message transmission in cancer cells and influence tumor behavior and be taken up by zebrafish in the aqueous environment (Konantz et al., 2019). Interestingly, using the zebrafish model, we can screen many compounds and identify chemicals that inhibit tumor growth and development. In addition, we can acquire circulating blood from adult zebrafish via cardiac puncture and measure the serum drug concentration by mass spectrometry (Yan et al., 2019). We can thus establish a correlation between the serum drug concentration in zebrafish and the specific effective drug concentration of patients. However, different drugs have different solubilities in water and absorptive characteristics into fish from water. Therefore, some drugs that are more hydrophobic can be dissolved in water by either promoting solvents or combining the drugs with nanomaterials (Wang et al., 2010; Evensen et al., 2016; Li et al., 2017; Sieber et al., 2017; Garcia-Caballero et al., 2018). Even some protein-based drugs or nanodrug carriers can be injected directly into the blood circulation via microinjection (Evensen et al., 2016; Wu et al., 2017, 2020).

In addition to its use in conventional drug screening, the zebrafish model is being applied to evaluate the effectiveness of radiotherapy (Gnosa et al., 2016; Costa et al., 2020a). Furthermore, zebrafish can be used with fluorescent labeling to experimentally confirm that various carriers, such as bacteria (which enhance antitumor effects) can be absorbed into the body (Xie et al., 2018; Chen et al., 2019; Shi et al., 2019). For instance, researchers marked cancer cells with Dir and labeled *Escherichia coli* strain Nissle 1917 (EcN) with GFP, and using confocal laser scanning microscopy, we could visually identify the relative position of EcN in the zebrafish tumor model (Shi et al., 2019).

## The Zebrafish Model in Studies of the Tumor Microenvironment

The tumor microenvironment plays an essential role in tumor formation, growth, metastasis and development (Chapman et al., 2014; Astell and Sieger, 2019; de Oliveira et al., 2019; Giallongo et al., 2019; Gómez-Abenza et al., 2019; Liu et al., 2019; Sun et al., 2019). Zebrafish, as a tumor model, are becoming increasingly prominent in studying the tumor microenvironment (Liu and Leach, 2011; Wang et al., 2015; Liu et al., 2017, 2019; Astell and Sieger, 2019; Giallongo et al., 2019; Gómez-Abenza et al., 2019; Sun et al., 2019). First, cancer cells can be implanted into different positions in zebrafish that provide distinct tumor microenvironments. Another example shows that, using a *plin2*-tdTomato transgenic line that shows the location of the deposits of subcutaneous fat, researchers can identify different zebrafish anatomy positions with distinct adipocyte fat pad contents (Zhang et al., 2018). Other groups transplanted a melanoma cell line into the ventral and dorsal subcutaneous fat pads of fish and verified the effect of adipocytes on melanoma metastasis (Zhang et al., 2018). In another study, investigators successfully injected three human liver cancer cell lines into the yolk sac of *ache*^−/−^ zebrafish and found that acetylcholine, one of the molecules found in the tumor microenvironment, facilitates cancer cell growth (Avci et al., 2018). In another experiment on tumor cells implanted into the yolk sac, researchers discovered that the hypoxic environment of the yolk sac may simulate the tumor microenvironment of some human tumors due to the lack of vascularization. They further found that the proliferation of primary effusion lymphoma cells in xenografts requires a hypoxic environment to stimulate eukaryotic translation initiation factor 4E2 (elF4E2) (Pringle et al., 2019).

**Figure 2:**
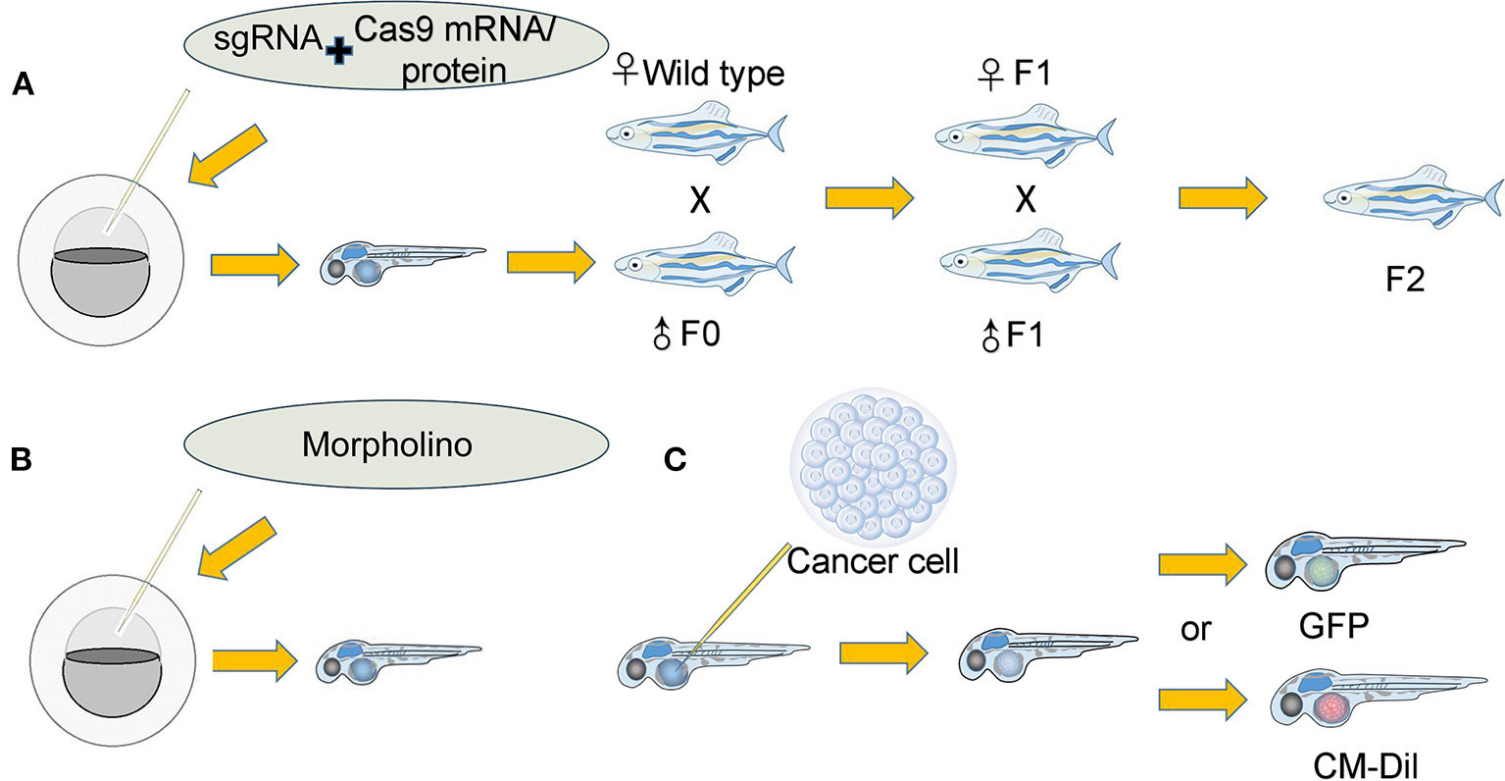
Establishment of the tumor microenvironment in the zebrafish xenograft model. **(A)**: (I) A single-guide RNA (sgRNA) was coinjected along with Cas9 mRNA/protein into either the yolk or cell of single-cell stage embryos. The coinjected embryos grew into founder fish. (II) The founder fish were then outcrossed to *wild-type* siblings to generate heterozygous F1 fish. Fish lacking the correct genotype (as identified by fluorescence PCR and sequencing) were eliminated. (III) The F1 fish carrying the correct mutations were then crossed to generate F2 progeny. F2 offspring will provide an optimal tumor microenvironment for researchers. **(B)**: Morpholino knockdown of specific genes in zebrafish embryos directly provides the tumor microenvironment. **(C)**: (I) Cancer cells that were either expressing GFP or labeled with other fluorescent dyes were implanted into zebrafish. (II) Various indexes of cancer cells were observed and recorded.

Transgenic zebrafish lines in which the expression of a specific gene is inhibited or overexpressed can also be used to study the tumor microenvironment (Figure 2) (Bedell et al., 2012; Jao et al., 2013; Irion et al., 2014; Sung et al., 2014; Kim et al., 2017) by providing a unique tumor microenvironment and to further explore the relationship between tumor development and the tumor microenvironment (Figure 2). The premise is that the gene signaling we explore is conserved between zebrafish and humans. There is a strong advantage in the zebrafish model because the zebrafish genome was revealed to have exceedingly high homology with the human genome (Burgess, 2013; Howe et al., 2013). For instance, researchers discovered that zebrafish *cxcr4* (*cxcr4b*) is highly expressed in neutrophils and controls neutrophil number and motility and that neutrophils interact with cancer cells to initiate early metastatic events. MDA-MB-231-B breast tumor cells were injected into the circulation of *wild-type* zebrafish and cxcr4b^−/−^ zebrafish embryos at 2 dpf. The researchers found that the tumor cells in *cxcr4b*^−/−^ zebrafish embryos did not influence neutrophil speed or distance traveled relative to *wild-type* siblings. In this experiment, GFP-labeled neutrophils were easily detected via confocal microscopy. In conclusion, researchers have proposed that *cxcr4b* signaling supports interactions between neutrophils and tumor cells and drives early tumor metastasis (Tulotta et al., 2019). In another experiment, liver cancer cells implanted into *ache*^−/−^ mutant zebrafish exhibited stronger growth and metastasis abilities than those implanted into *wild-type* siblings (Avci et al., 2018). Additionally, glioma cells implanted into *irf8*^−/−^ transgenic zebrafish that lack microglia and wild-type zebrafish treated with CSF-1 inhibitor (a pharmacological means of decreasing microglia) have demonstrated a unique role for zebrafish microglia in facilitating human glioma cell growth (Hamilton et al., 2016). Similarly, researchers separately created CRISPR-mutant zebrafish in which the function of *EDN3* or *ECE2* in the tumor microenvironment was lost, with both mutations exerting a severe effect on melanocyte development. Then, researchers transplanted melanoma cells into *wild-type, EDN3-*, or *ECE2*-deficient transgenic zebrafish and verified that variation in the tumor microenvironment exerts a tremendous impact on melanoma development and metastasis (Kim et al., 2017). The tumor microenvironment is established by a change in gene expression in the short term but can also affect tumor behaviors. Morpholino injection is also a method used to alter gene expression (Figure 2) (Gamble et al., 2018). Using this method, researchers found that the change in laminin alpha 5 expression in the tumor microenvironment influenced glioblastoma microtumor formation and invasion (Gamble et al., 2018). The tumor microenvironment provided by the zebrafish model possesses a unique advantage.

The tumor immune microenvironment is also critical in cancer development and metastasis. Similar to humans, zebrafish also have a generally defined immune system (Lam et al., 2002; Danilova et al., 2004; Langenau et al., 2004; Langenau and Zon, 2005; Giallongo et al., 2016; de Oliveira Mann et al., 2019). By exploiting this characteristic, scientists showed that mesenchymal stromal cells are involved in the tumor immune microenvironment transformation of multiple myeloma (Giallongo et al., 2019). The innate immune microenvironment includes not only the neutrophils mentioned above but also macrophages. Some research groups generated a *myd88*^w187^ mutant and *pu.1/spi1b*^fh509^ mutant zebrafish strain, which affect the number and function of macrophages, respectively, in the zebrafish body (Roh-Johnson et al., 2017). It was further demonstrated that macrophages transfer cytoplasm to tumor cells and that this transfer correlates with increased tumor cell dissemination *in vivo* (Roh-Johnson et al., 2017).

## Discussions and Future Perspectives

The optical clarity of zebrafish allows us to accurately observe the shape and size of a tumor, the movement and morphology of a single cell of the tumor, interactions between cells, and various physical indicators related to tumor growth. Transgenic zebrafish with vascular-specific fluorescence allow the observation of tumor neovascularization, which is an important condition for tumor development. Zebrafish models of tumor metastasis and intracranial orthotopic tumors as well their unique pharmacokinetics compensate for the limitations of tumor models in mice. The unique zebrafish tumor microenvironment and changes in the expression of certain genes provide an emerging and useful animal model for studying the effect and mechanism of the tumor microenvironment on tumor cells *in vivo*. These features of the zebrafish tumor model should be innovatively applied to tumor research. The tumor-targeting ability of drugs and the *in vivo* biodistribution of a drug are always the focuses of anticancer drug research. Optical transparency and easy real-time monitoring make zebrafish an ideal model organism for observing the distribution of a drug. Additionally, the switch of metastatic phenotypes is also observed when the tumor microenvironment changes. For instance, by moving cancer cells to more restrictive tumor microenvironments, we observed that breast cancer cells switched from a metastatic phenotype, which depends on integrin adhesion and actin polymerization, to a tubulin-dependent metastatic phenotype (Balzer et al., 2012; Stroka and Konstantopoulos, 2014). This implies that the physical properties of the cellular microenvironment, such as the proportions of matrix constituents, concentration of matrix proteins or levels of cytokines, can induce switching between migratory phenotypes (Stuelten et al., 2018). The zebrafish model, which offers a direct visual model of metastasis, may help answer some questions, such as which cellular or microenvironmental parameters trigger transformation and the results of switching migratory phenotypes (Stuelten et al., 2018).

One future direction in the use of zebrafish as a model organism of tumor growth and progression is to better mimic the tumor environment of the human body. An interesting study reported the generation of mouse-zebrafish hematopoietic tissue-derived chimeric embryos (Parada-Kusz et al., 2018). This finding implies that we are likely to reconstruct a more human-like tumor microenvironment in the zebrafish model (Vinothkumar et al., 2019). There are still many needs to be addressed in studies of the tumor immune microenvironment, such as the adaptive immune microenvironment. Although zebrafish possess regenerative abilities, spinal cord injury may lead to massive losses of innate immune cells (Tsarouchas et al., 2018). Hence, when we complete the microinjections, we should exclude zebrafish with spinal damage.

With the development and application of robotics to inject zebrafish embryos, the limitation of inconsistencies when generating models has been addressed (Spaink et al., 2013). Although the issue of temperature differences between the xenograft and host was not addressed, there were studies reporting 34–35°C as a compromise allowing growth of both human cancer cells and fish (Haldi et al., 2006; Nicoli et al., 2007; Smith et al., 2013; Bentley et al., 2015; Fazio et al., 2020). These difficulties, including inconsistencies in protocols between different laboratories and a lack of reproducibility, also limit the widespread use of zebrafish. *To overcome these* limitations, it is necessary to strengthen the contact and information sharing between labs. In conclusion, although no model is perfect, the zebrafish model has unique advantages and even plays an irreplaceable role in some respects.

## Author Contributions

RJ concepted and designed the review. XC and YL drafted the manuscript. TY drawn the figures. All the authors participated in writing and final approval of the manuscript and are responsible for all aspects of the work.

## Conflict of Interest

The authors declare that the research was conducted in the absence of any commercial or financial relationships that could be construed as a potential conflict of interest.
